# Complete genome sequence of *Brevibacterium luteolum* strain DMY-1, a styrene-degrading bacterium isolated from livestock wastewater

**DOI:** 10.1128/mra.00954-25

**Published:** 2025-10-13

**Authors:** Byung-Gon Ryu, Young Ho Nam, Young Taek Oh, Kook-Il Han

**Affiliations:** 1 Using Technology Development Department, Nakdonggang National Institute of Biological Resources (NNIBR), Sangju-si, Gyeongsangbuk-do, Republic of Korea; University of Wisconsin-Madison, Madison, Wisconsin, USA

**Keywords:** *Brevibacterium luteolum*, odor reducing bacterium, styrene

## Abstract

The complete genome sequence of *Brevibacterium luteolum* strain DMY-1, which was isolated from the livestock wastewater, has the ability to degrade styrene. Its genome consists of a circular 3.07 Mbp chromosome with a G+C content of 67.3% and encompasses 2,757 protein-coding sequences.

## ANNOUNCEMENT

*Brevibacterium luteolum* is a gram-positive bacterium with potential applications in various industrial processes. This species has been reported to produce keratin-degrading enzymes (1), biosurfactants (2), and alkaline proteases (3). Extracts from *B. luteolum* have also shown antiproliferative activity against hepatocellular carcinoma cell lines (4). Strain DMY-1 was isolated during a study on microorganisms for odor reduction.

Here, we announce the genome sequence of strain *B. luteolum* DMY-1, isolated from livestock wastewater collected at a pig farm in Sangju City, Republic of Korea (36°25’N and 128°18’E). A total of 5 g of slurry was inoculated into minimal salt medium (MSM, per liter: 3.5 g K₂HPO₄, 1.5 g KH₂PO₄, 0.27 g MgSO₄, 1 g NH₄Cl, 0.03 g Fe₂(SO₄)₃·7H₂O, and 0.03 g CaCl₂) containing 200 ppm styrene and incubated at 30°C with shaking at 150 rpm for 2 weeks under aerobic conditions for enrichment, resulting in diverse colonies. Briefly, *B. luteolum* strain DMY-1 was enriched in MSM medium at 30°C with shaking at 150 rpm for 2 weeks under aerobic conditions. Colonies were purified on tryptic soy agar (TSA) after serial dilution and were subcultured three times until a pure colony was obtained.

For genomic analysis, strain DMY-1 was grown on TSA at 30°C for 3 days. Genomic DNA was extracted using the Maxwell RSC Tissue DNA kit (Promega, USA). For long-read sequencing, DNA was sheared with a g-TUBE (Covaris, USA), and libraries were prepared using the SMRTbell Express kit (PacBio). Sequencing was performed on the PacBio Sequel II platform. To complement this, Illumina short-read sequencing was performed using the TruSeq Nano DNA kit and the NovaSeq 6000 platform (Illumina, USA).

PacBio sequencing yielded 81,731 reads (N50: 9,621 bp), and Illumina sequencing generated 10,745,004 paired-end reads (2×150 bp). *De novo* assembly of long reads was conducted with HGAP4 (5). Short reads were quality-trimmed with Trimmomatic v0.38 (6) and polished using Pilon v1.22 (7). Genome completeness was assessed with BUSCO v5.1.3 (8). Annotation was performed with Prokka v1.14.6 (9) and the NCBI Prokaryotic Genome Annotation Pipeline v6.8 with GeneMarkS-2+ (10). Circular genome visualization was generated using Circos v0.69.6 (11). Default parameters were used except where otherwise noted.

The final assembly consisted of a single circular chromosome of ~3.07 Mbp with a GC content of 67.3% (Table 1). The genome harbors genes involved in styrene degradation, including *hcnC*_1–2 and *hcnA*. Genes related to sulfur compound reduction (*cysK*1, *cysE*, *tpx*, and *sseA*), trimethylamine degradation (*gcvP*, *gcvT*, and *gcvH*_1–2), and volatile amine metabolism (*mshA*_1–4) were also identified, suggesting potential applications in odor mitigation.

**TABLE 1:** Summary of the assembly and annotation statistics for *B. luteolum* DMY-1

Parameter data	Result
Genetic element	Chromosome
Length (bp)	3,071,407
GC content (%)	67.3
No. of coding sequences	2,757
No. of rRNAs	6
No. of tRNAs	45
Sequencing depth (×)	221
GenBank accession no.	CP184789

## Data Availability

The genome sequence and raw sequencing reads for strain DMY-1 were deposited under GenBank accession number CP184789, BioProject accession number PRJNA1232853, BioSample accession number SAMN47251453 and Sequence Read Archive (SRA) accession numbers SRR32725459 and SRR32725458. *Brevibacterium luteolum* strain DMY-1 has been deposited in the Korean Collection for Type Cultures under accession number KCTC 15890BP.
